# Generation and analysis of knock-in mice carrying pseudohypoaldosteronism type II-causing mutations in the *cullin 3* gene

**DOI:** 10.1242/bio.013276

**Published:** 2015-10-21

**Authors:** Yuya Araki, Tatemitsu Rai, Eisei Sohara, Takayasu Mori, Yuichi Inoue, Kiyoshi Isobe, Eriko Kikuchi, Akihito Ohta, Sei Sasaki, Shinichi Uchida

**Affiliations:** Department of Nephrology, Graduate School of Medical and Dental Sciences, Tokyo Medical and Dental University, 1-5-45 Yushima, Bunkyo, Tokyo 113-0034, Japan

**Keywords:** Cullin 3, PHAII, Hypertension

## Abstract

Pseudohypoaldosteronism type II (PHAII) is a hereditary hypertensive disease caused by mutations in four different genes: *with-no-lysine kinases* (*WNK*) *1* and *4*, *Kelch-like family member 3* (*KLHL3*), and *cullin 3* (*Cul3*). Cul3 and KLHL3 form an E3 ligase complex that ubiquitinates and reduces the expression level of WNK4. PHAII-causing mutations in *WNK4* and *KLHL3* impair WNK4 ubiquitination. However, the molecular pathogenesis of PHAII caused by *Cul3* mutations is unclear. In cultured cells and human leukocytes, PHAII-causing *Cul3* mutations result in the skipping of exon 9, producing mutant Cul3 protein lacking 57 amino acids. However, whether this phenomenon occurs in the kidneys and is responsible for the pathogenesis of PHAII *in vivo* is unknown. We generated knock-in mice carrying a mutation in the C-terminus of intron 8 of *Cul3*, c.1207−1G>A, which corresponds to a PHAII-causing mutation in the human *Cul3* gene. Heterozygous *Cul3*^G(−1)A/+^ knock-in mice did not exhibit PHAII phenotypes, and the skipping of exon 9 was not evident in their kidneys. However, the level of Cul3 mRNA expression in the kidneys of heterozygous knock-in mice was approximately half that of wild-type mice. Furthermore, homozygous knock-in mice were nonviable. It suggested that the mutant allele behaved like a knockout allele and did not produce Cul3 mRNA lacking exon 9. A reduction in Cul3 expression alone was not sufficient to develop PHAII in the knock-in mice. Our findings highlighted the pathogenic role of mutant Cul3 protein and provided insight to explain why PHAII-causing mutations in *Cul3* cause kidney-predominant PHAII phenotypes.

## Abstract (precis)

**Summary:** A knock-in mutation in intron 8 of *Cul3* in mice led to decreased Cul3 protein expression. Decreased Cul3 protein expression alone did not cause pseudohypoaldosteronism type II (PHAII).

## INTRODUCTION

Pseudohypoaldosteronism type II (PHAII) is a hereditary disease characterized by hypertension, hyperkalemia, and metabolic acidosis (Gordon, 1986). Activation of the thiazide-sensitive Na-Cl cotransporter (NCC) in the distal convoluted tubules is the main pathogenesis of this disease because all of its phenotypes are sensitive to thiazide treatment (Mayan et al., 2002). Mutations in *with-no-lysine kinase 1* (*WNK1*) and *with-no-lysine kinase* *4* (*WNK4*) were reported to be responsible for PHAII in 2001 (Wilson et al., 2001). However, at that time, the connection of WNK to NCC was totally unknown. We generated a knock-in mouse model of PHAII harboring a PHAII-causing *WNK4* mutation (D561A, equivalent to D564A in human), and discovered a novel signaling pathway from WNK to NCC, namely WNK–oxidative stress-responsive 1 (OSR1)/ste20-like proline/alanine-rich kinase (SPAK)–NCC phosphorylation signaling (Chiga et al., 2011; Chu et al., 2013; Takahashi et al., 2014; Yang et al., 2007). Although this signaling pathway was usually regulated according to salt intake in wild-type mice, OSR1, SPAK, and NCC phosphorylation were constitutively active in *WNK4* (D561A) PHAII model mice (Chiga et al., 2011). PHAII-causing mutations in *WNK4*, including D564A, were clustered in a so-called ‘acidic domain’, where the amino acid sequence is highly conserved among all WNKs (Wilson et al., 2001).

In 2012, *Kelch-like family member 3* (*KLHL3*) and *cullin 3* (*Cul3*) were newly identified as genes responsible for PHAII by an exome sequencing strategy (Boyden et al., 2012; Louis-Dit-Picard et al., 2012). KLHL3 is a BTB domain protein and forms a complex with Cul3 (Ji and Prive, 2013). We demonstrated that *KLHL3* bound to *Cul3* via its BTB domain and formed an E3 ligase for WNK4, and that PHAII-causing mutations in *WNK4* and *KLHL3* disrupted the formation of the WNK4–KLHL3–Cullin3 complex and impaired the ubiquitination and degradation of WNK4 (Mori et al., 2013; Ohta et al., 2013; Wakabayashi et al., 2013). Increased WNK4 activated downstream OSR1/SPAK–NCC signaling. In contrast to these mutations in *WNK4* and *KLHL3*, the *Cul3* mutations reported to date are *de novo* mutations, and are mostly present not in the coding exons, but in the introns around exon 9 (Boyden et al., 2012).

Studies of blood samples collected from patients with PHAII caused by *Cul3* mutations revealed that *Cul3* mutations resulted in the loss of exon 9 during splicing. This may lead to the production of a mutant Cul3 protein with a 57-amino acid deletion (Δ403–459) (Boyden et al., 2012; Tsuji et al., 2013). A recent study (McCormick et al., 2014) revealed that this mutant Cul3 protein was more neddylated and activated than wild-type Cul3 in cultured cells, and consequently degraded KLHL3 protein. Nephron-specific *Cul3* knockout mice exhibited increased levels of WNK proteins and phosphorylated NCC (McCormick et al., 2014). Although these data suggested that Cul3 was involved in WNK degradation in the kidney and that the function of mutant Cul3 protein was altered, the molecular pathogenesis of PHAII caused by *Cul3* mutations remains unclear. As mentioned earlier, Cul3 is expressed ubiquitously and forms E3 ligase complexes with various adaptor proteins in addition to KLHL3. Does the skipping of exon 9 occur and is the deletion mutant present in the nephron segments where WNK and KLHL3 reside? Why do mutations in these proteins cause kidney predominant PHAII phenotypes?

To address these questions, the generation and analysis of knock-in mouse models, carrying the same pathogenic mutations present in human patients with PHAII, represents the best approach. In this study, we generated two different lines of *Cul3* knock-in mice carrying mutations in the C terminus of intron 8, c.1207−1G>A (G(−1)A) and c.1207−6T>G (T(−6)G), which correspond to the two reported PHAII-causing *Cul3* mutations in humans (Boyden et al., 2012; Tsuji et al., 2013). Unfortunately, we were unable to detect the loss of exon 9 during splicing in these *Cul3* knock-in mice. However, the analysis of heterozygous *Cul3*^G(−1)A/+^ knock-in mice revealed that Cul3 expression level in the kidneys was reduced to approximately half that of wild type mice. It suggested that the mutant allele did not result in the skipping of exon 9, but rather behaved like a knockout allele. We studied the mice further to clarify whether a reduction in Cul3 expression played a role in the development of PHAII. The results may provide a valuable insight for the role of mutant Cul3 protein with a 57-amino acid deletion (Δ403–459) in the pathogenesis of PHAII.

## RESULTS

### Generation of knock-in mice carrying pseudohypoaldosteronism type II-causing mutations in the *cullin 3* gene

**Fig. 1.**
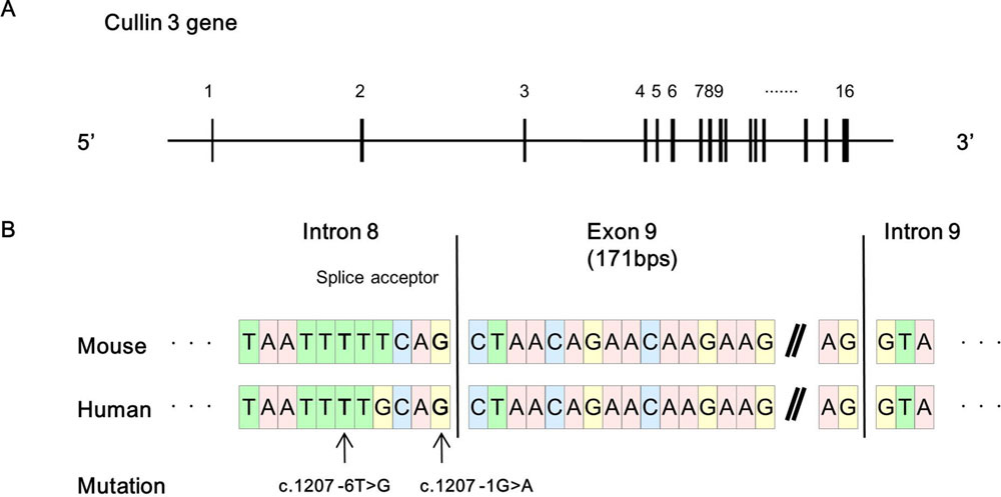
**Sequence homology between human and mouse *cullin 3*.** (A) Exon–intron structure of mouse c*ullin 3* (*Cul3*; NCBI Reference Sequence: NC_000067.6). Boxes represent exons. (B) Comparison of the sequence homology between mouse *Cul3* (NCBI Reference Sequence: NC_000067.6) and human *Cul3* (NCBI Reference Sequence: NG_032169.1). The sequence of *Cul3* is highly conserved between the species; indeed, the length of exon 9 is 171 base pairs in both species. The indicated mutations are pseudohypoaldosteronism Type II-causing mutations in *Cul3* located within the splice acceptor site in intron 8.

**Fig. 2.**
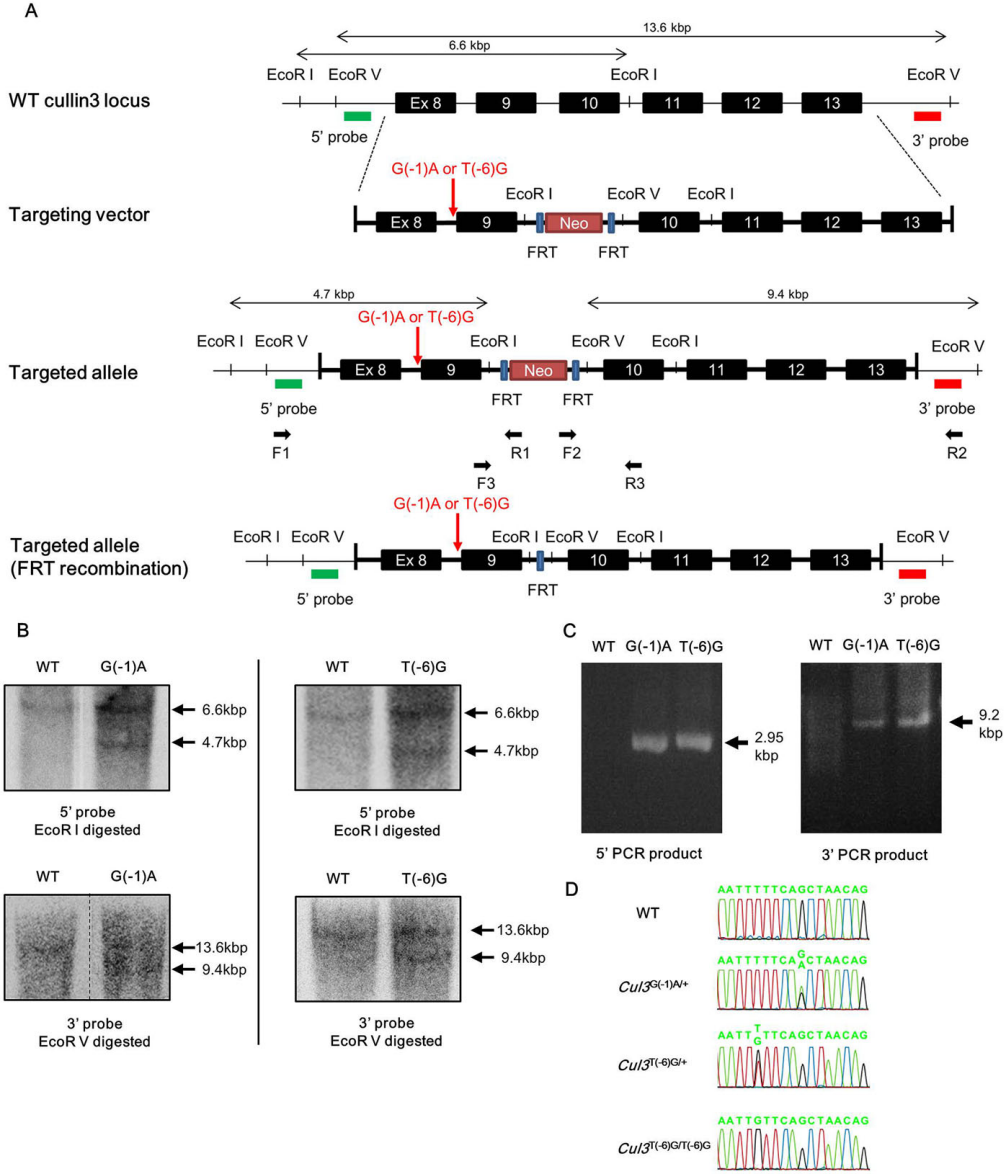
**Targeting strategy for generating *cullin 3* knock-in mice.** (A) Wild-type *cullin 3* (*Cul3*) locus, the targeting construct, and the targeted locus before and after FLPe recombination. Primers F1, F2, F3, R1, R2, and R3 are shown on the targeted locus. Probes used for southern blotting are shown as 5′ and 3′ probes. (B) Verification of homologous recombination by southern blotting. Genomic DNA was digested with *EcoR*I, and southern blotting was performed with the 5′ probe. The 6.6-kbp band was generated from the wild-type allele and the 4.7-kbp band was generated from the mutant allele (top). Genomic DNA was digested with *EcoR*V, and southern blotting was performed with the 3′ probe. The 13.6-kbp band was generated from the wild-type allele and the 9.4-kbp band was generated from the mutant allele (bottom). (C) Polymerase chain reaction (PCR)-verification of homologous recombination using the genomic DNA of selected embryonic stem cell (ESC). The locations for primers F1, F2, R1, and R2 are shown in A. The 2.95-kbp (5′-side PCR) and 9.2-kbp (3′-side PCR) bands were generated from the mutant allele. Primer sets were designed to prevent amplification of the wild-type Cul3 gene. WT: host ES cells. (D) Direct sequencing of the PCR product covering the mutation site. WT: wild type. G(−1)A: *Cul3* c.1207 −1G>A. T(−6)G: *Cul3* c.1207 −6T>G.

PHAII-causing mutations in *Cul3* are known to cluster around exon 9 of the *Cul3* gene, and some are located within the splice acceptor site in intron 8 (Boyden et al., 2012). We compared the homology of the *Cul3* genome sequences between *Homo sapiens* and *Mus musculus*. Exon 9 of *Cul3* was composed of 171 bp in both species, and its sequence was highly conserved (Fig. 1). Of the known PHAII-causing mutations, we selected two mutations, c.1207−1G>A (G(−1)A) and c.1207−6T>G (T(−6)G), to generate the knock-in mice. We designed targeting vectors to introduce the mutations into *Cul3*, as shown in Fig. 2. We confirmed homologous recombination by PCR, southern blotting, and sequencing of the mutation site in ES cell lines. Chimeric mice were generated by injection of the ES cells into blastocysts. Chimeric male offspring were mated with C57BL/6 females, yielding heterozygous mice. Next, we crossed the heterozygous knock-in mice with FLPe recombinase-expressing transgenic mice to remove the FRT-flanked neomycin cassette. We successfully generated F1 heterozygous knock-in mice carrying the knocked-in allele (*Cul3*^G(−1)A^ or *Cul3*^T(−6)G^) and wild-type allele: namely *Cul3*^G(−1)A/+^ and *Cul3*^T(−6)G/+^ mice.

### *Cullin 3* expression in the knock-in mouse models

F1 heterozygous mice were intercrossed to obtain homozygous F2 mice. Among the first 100 progeny, no viable *Cul3*^G(−1)A/G(−1)A^ mice were obtained. *Cul3*^+/+^ and *Cul3*^G(−1)A/+^ mice were obtained at the expected frequencies. Conversely, *Cul3*^T(−6)G/T(−6)G^, *Cul3*^T(−6)G/+^, and *Cul3*^+/+^ mice were obtained at the expected frequencies.

**Fig. 3.**
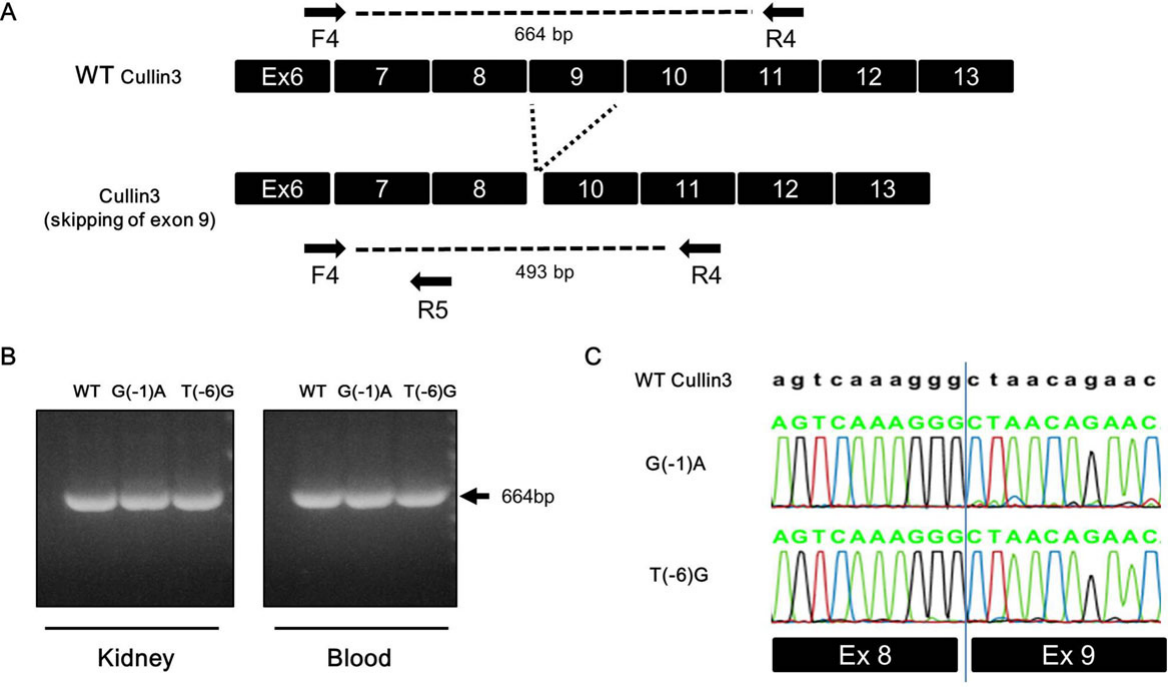
**Reverse-transcription polymerase chain reaction of the spliced RNA.** Reverse-transcription (RT) polymerase chain reaction (PCR) of the spliced RNA was performed. (A,B) PCR using a primer set (F4 and R4) flanking exon 9. Wild-type *Cullin 3* (*Cul3*) cDNA produced a single product that included exon 9 (664 bp). If exon 9 of CUL3 was skipped, the expected PCR product would be 493 bp. We detected only the bands including exon 9. (C) Representative RT-PCR sequences. Complementary DNA with properly spliced junctions between exons 8 and 9 was confirmed in both types of knock-in mice. WT: wild type. G(−1)A: *Cul3* c.1207 −1G>A knock-in mice. T(−6)G: *Cul3* c.1207 −6T>G knock-in mice.

We prepared mRNA from the blood and kidneys of these mice and performed reverse-transcription PCR to investigate *Cul3* expression from the mutant allele. In the blood and kidneys of *Cul3*^G(−1)A/+^ mice, we detected wild-type *Cul3* mRNA, but did not detect mutant *Cul3* mRNA from which exon 9 was expected to be lost during splicing (Fig. 3). Similarly, we detected only wild-type *Cul3* mRNA in the blood and kidneys of *Cul3*^T(−6)G/T(−6)G^ mice.

**Fig. 4.**
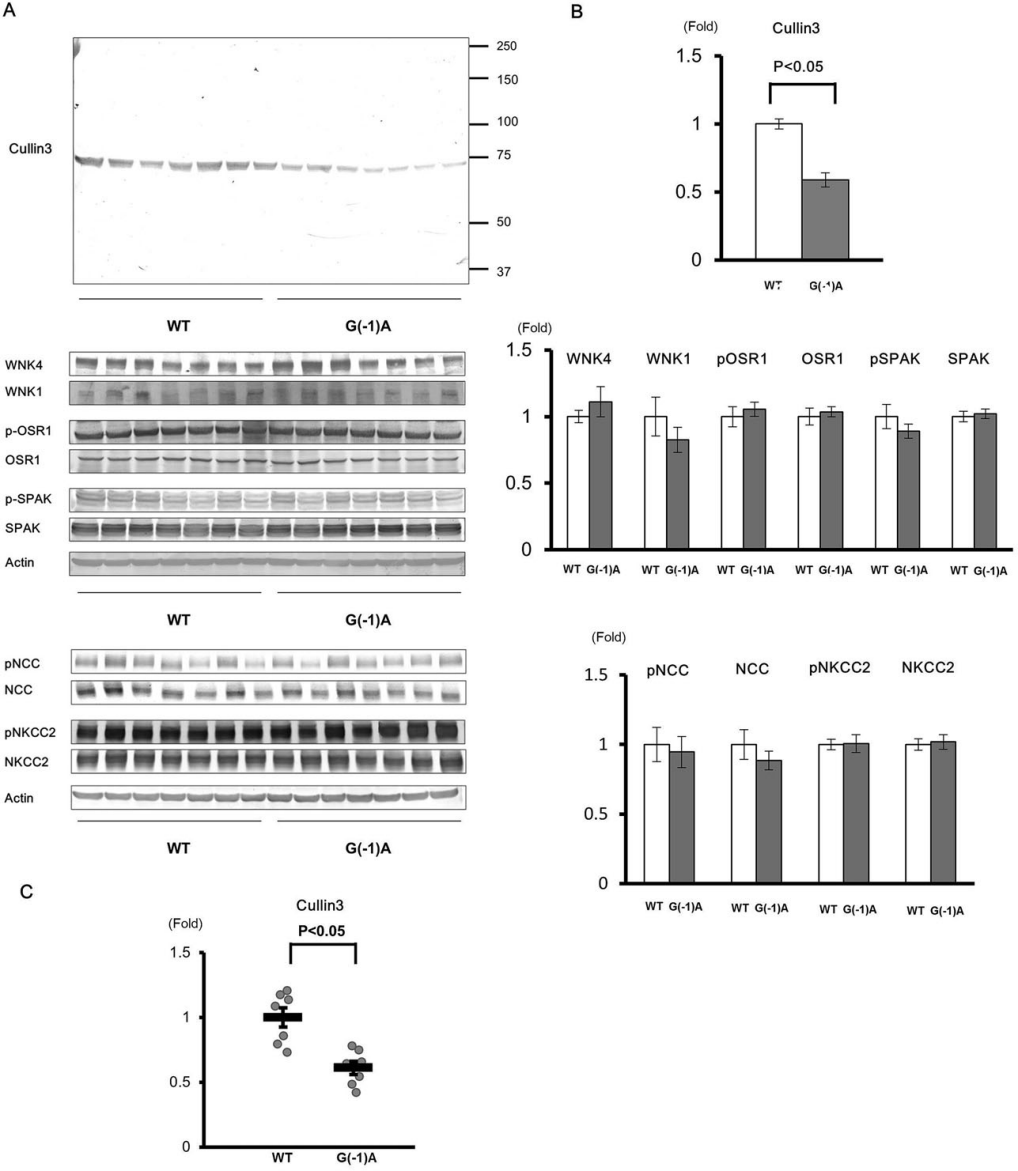
**Expression levels of cullin 3 protein and mRNA and proteins of the WNK–OSR1/SPAK–NCC phosphorylation signaling cascade in the kidneys of *Cullin 3*^G(−1)A/+^ knock-in mice.** (A) Immunoblots of proteins of the WNK–OSR1/SPAK–NCC signaling cascade in the kidneys of wild-type (WT) and *cullin 3* (*Cul3*)^G(−1)A/+^ heterozygous knock-in mice. (B) Densitometry analysis. Values are expressed as a ratio of the average signal in WT mice. Cul3 expression levels in *Cul3*^G(−1)A/+^ heterozygous knock-in mice were approximately half that of WT mice. There were no significant differences in proteins expression levels of the WNK–OSR1/SPAK–NCC phosphorylation signaling cascade between *Cul3*^G(−1)A/+^ heterozygous knock-in and WT mice. (C) Quantitative polymerase chain reaction (PCR) analysis of *Cul3* mRNA levels. SYBR Green quantitative PCR was used to quantify mRNA levels in the kidneys of WT mice (*n*=7) and *Cul3*^G(−1)A/+^ mice (*n*=7). WT: wild type. G(−1)A: *Cul3*^G(−1)A/+^. *P<0.05 compared with wild-type mice, data presented as mean±s.e.m.

**Fig. 5.**
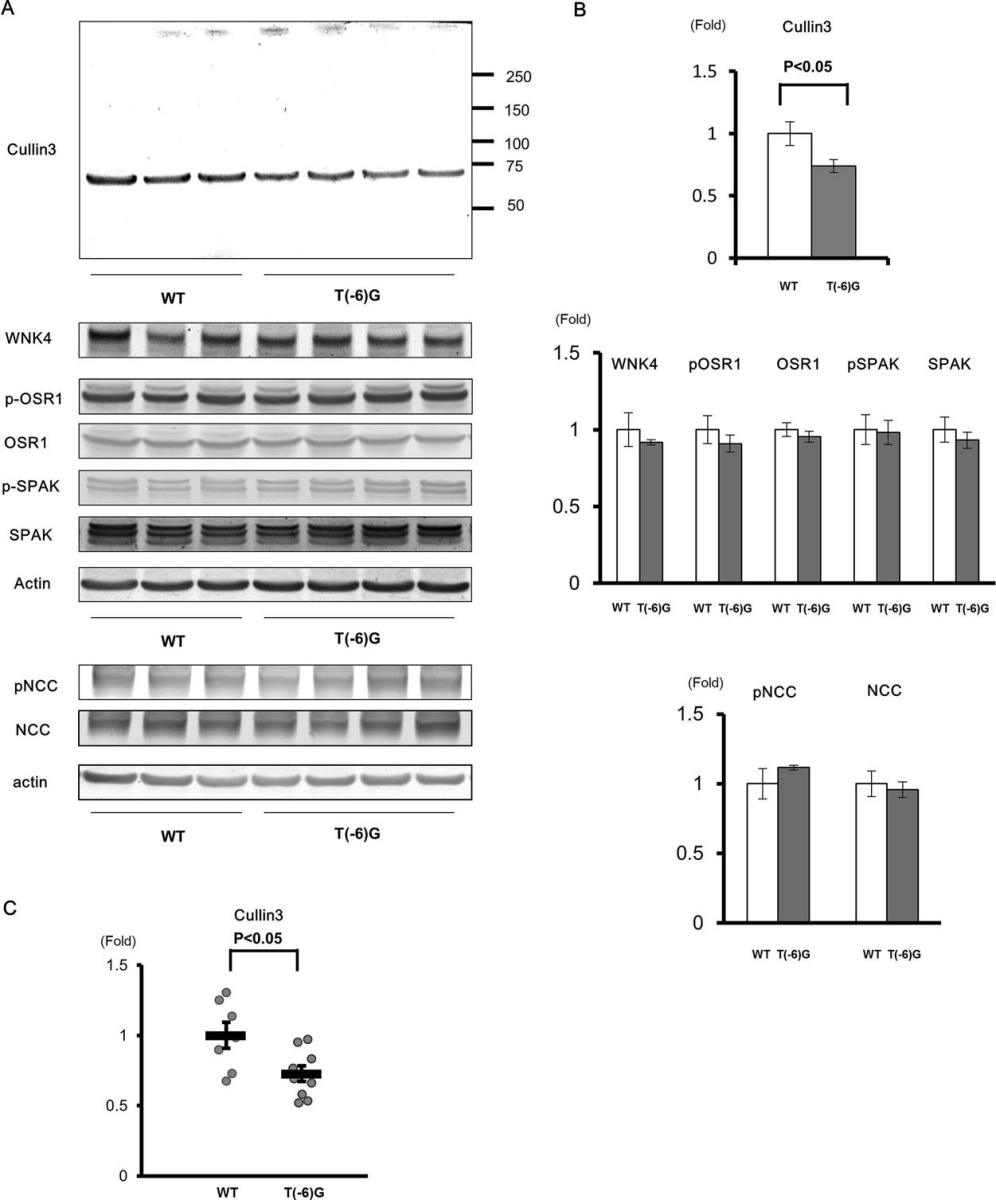
**Expression levels of cullin 3 protein and mRNA and proteins of the WNK-OSR1/SPAK-NCC phosphorylation signaling cascade in the kidneys of *cullin 3*^T(−6)G/T(−6)G^ knock-in mice.** (A) Immunoblots of proteins of the WNK–OSR1/SPAK–NCC signaling cascade in the kidneys of wild-type (WT) and *cullin 3* (*Cul3*) ^T(**−**6)G/T(**−**6)G^ homozygous knock-in mice. (B) Densitometry analysis. Values are expressed as the ratio of the average signal in WT mice. The level of Cul3 protein expression in *Cul3*^T(**−**6)G/T(**−**6)G^ mice was three-quarters that of WT mice. There were no significant differences in proteins expression levels of the WNK–OSR1/SPAK–NCC phosphorylation signaling cascade between *Cul3*^T(**−**6)G/T(**−**6)G^ and WT mice. (C) Quantitative polymerase chain reaction (PCR) analysis of Cul3 mRNA levels. SYBR Green quantitative PCR was used to quantify mRNA levels in the kidneys of WT mice (*n*=7) and *Cul3*^T(**−**6)G/T(**−**6)G^ mice (*n*=9). WT: wild-type mice. T(−6)G: *Cul3*^T(**−**6)G/T(**−**6)G^ mice. *P<0.05 compared with WT mice, data presented as mean±s.e.m.

Quantitative reverse-transcription PCR revealed that the expression of wild-type *Cul3* mRNA in *Cul3*^G(−1)A/+^ mice was approximately half that of wild-type mice (Fig. 4C). Next, we analyzed protein extracts from the entire kidney without the nuclear fraction using western blotting. We used anti-Cul3 antibody raised against the N terminus of Cul3. We detected protein bands with the expected molecular size of wild-type Cul3, but did not detect any truncated forms of Cul3 (Fig. 4A). Nonetheless, wild-type Cul3 expression level in *Cul3*^G(−1)A/+^ mice was half that in wild-type mice (Fig. 4B). Conversely, quantitative reverse-transcription PCR revealed that the expression of wild-type *Cul3* mRNA in *Cul3*^T(−6)G/T(−6)G^ mice was approximately three-quarters that of wild-type mice. Cul3 protein expression level in *Cul3*^T(−6)G/T(−6)G^ mice was also approximately three-quarters that of wild-type mice (Fig. 5).

### Blood analysis and blood pressure measurements

**Fig. 6.**
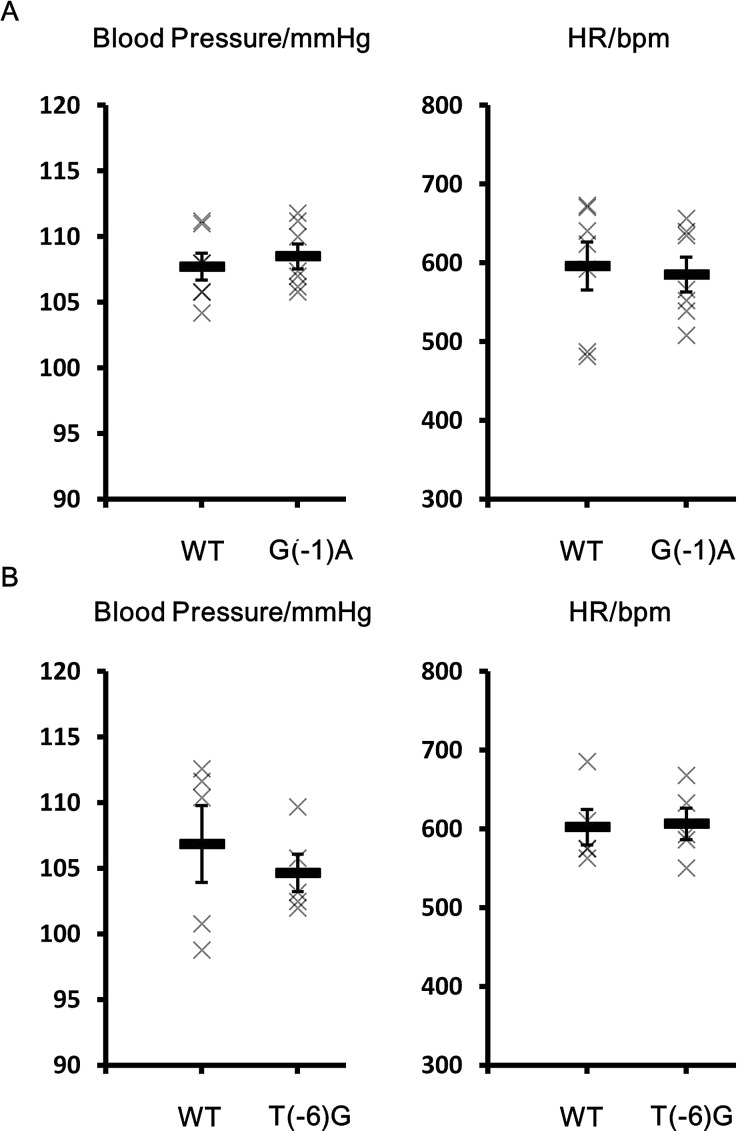
**Blood pressure and heart rate in wild-type, *cullin 3*^G(−1)A/+^ and *cullin 3*^T(−6)G/T(−6)G^ mice.** No significant differences in blood pressures or heart rate were evident between WT and *cullin 3* (*Cul3*)^G(**−**1)A/+^ mice or WT and *Cul3*^T(**−**6)G/T(**−**6)G^ mice. WT: wild type mice. G(−1)A: *Cul3*^G(**−**1)A/+^ mice. T(−6)G: *Cul3*^T(**−**6)G/T(**−**6)G^ mice. Data presented as mean±s.e.m.

We measured blood pressure in *Cul3*^G(−1)A/+^ mice using the tail-cuff method. No significant difference in blood pressure was evident between heterozygous *Cul3*^G(−1)A/+^ knock-in and wild-type mice (Fig. 6). We also examined the blood chemistry of *Cul3*^G(−1)A/+^ mice. Although the blood chloride level was slightly higher in wild-type mice, we detected no significant differences in any other parameter measured, including blood potassium levels and pH (Table 1). We also studied *Cul3*^T(−6)G/T(−6)G^ mice and found no significant difference in the blood chemistry or blood pressure between *Cul3*^T(−6)G/T(−6)G^ mice and wild-type mice. Thus, the blood chemistry and blood pressure-related characteristics of PHAII were not exhibited by the *Cul3* knock-in mice (Fig. 6, Table 2).

**Table 1.**
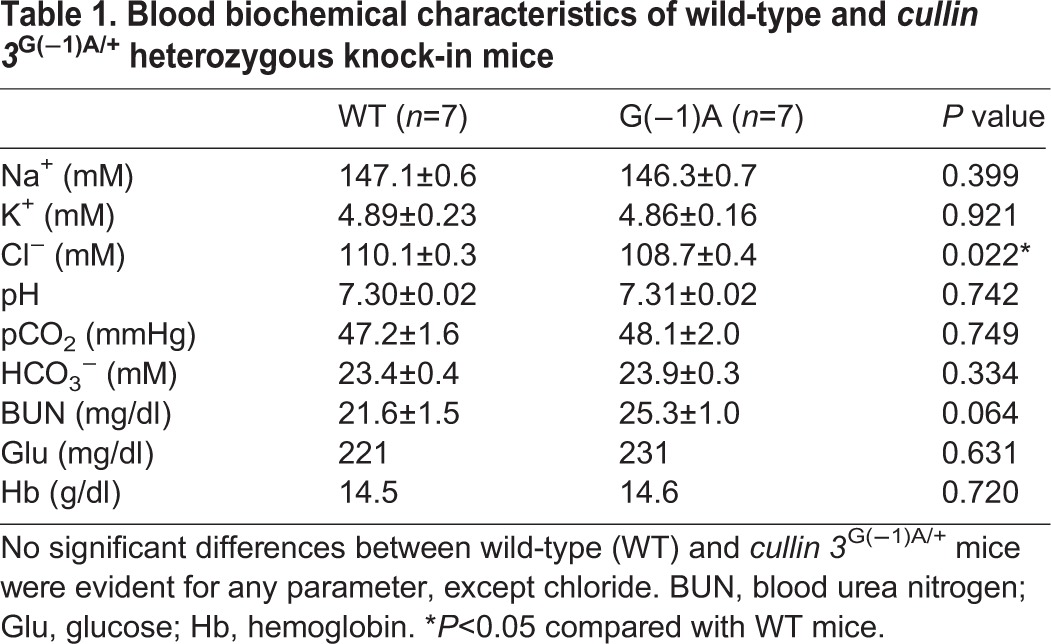
**Blood biochemical characteristics of wild-type and** ***cullin 3*****^G(−1)A/+^ heterozygous knock-in mice**

**Table 2.**
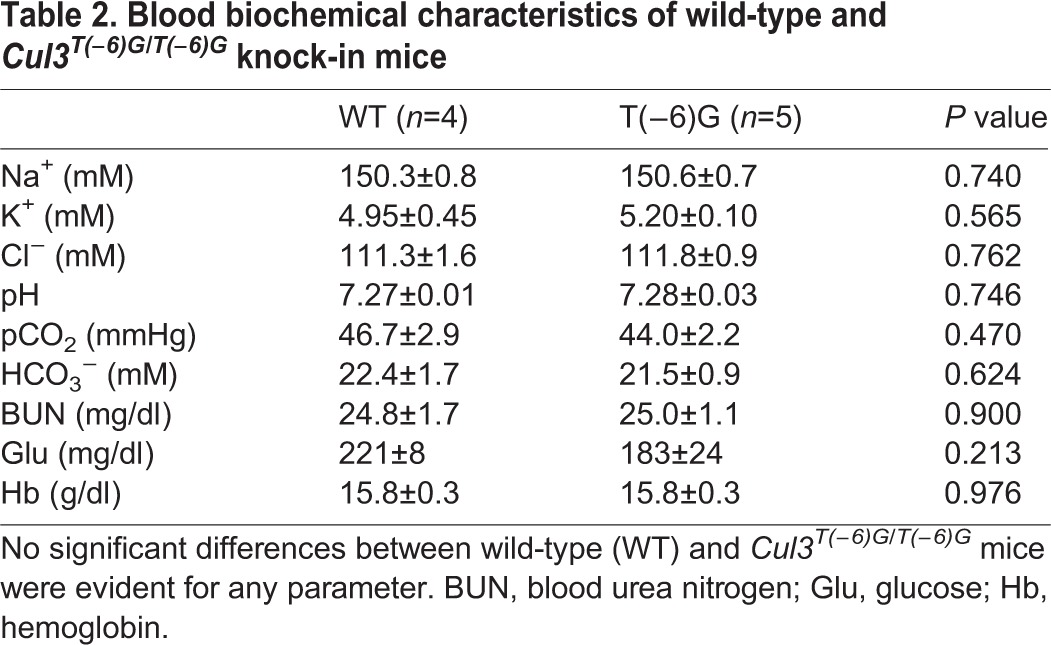
**Blood biochemical characteristics of wild-type and** ***Cul3^T(−6)G/T(−6)G^*** **knock-in mice**

### Expression of proteins associated with WNK–OSR1/SPAK–NCC signaling

Immunoblots of kidney samples from mice receiving a normal diet were performed. In heterozygous *Cul3*^G(−1)A/+^ knock-in mice, although Cul3 protein expression level in the kidney was about half that of wild-type mice, WNK4, WNK1, SPAK, OSR1, NCC, and NKCC2 expression levels in the kidney were not significantly altered by the reduction in Cul3. Similarly, no significant differences in phosphorylated SPAK, OSR1, NCC, and NKCC2 protein levels were evident between heterozygous *Cul3*^G(−1)A/+^ knock-in and wild-type mice (Fig. 4A,B). We also studied *Cul3*^T(−6)G/T(−6)G^ mice and found no significant difference in expression of proteins associated with WNK-OSR1/SPAK-NCC signaling (Fig. 5).

## DISCUSSION

Cullin–really interesting new gene (RING) ubiquitin ligases (CRLs) are the most prevalent class of E3 ubiquitin ligases (Hua and Vierstra, 2011; Petroski and Deshaies, 2005). CRL3s are subfamily of CRLs (Du et al., 1998). The molecular scaffold Cul3 and the RING protein RBX1 compose CRL3s, in combination with one of the BTB domain-containing proteins that act as substrate adaptors (Figueroa et al., 2005; Furukawa et al., 2003; Zheng et al., 2002). These complexes mediate the ubiquitination and subsequent proteasomal degradation of target proteins. Previous studies (Singer et al., 1999; Venugopal and Jaiswal, 1998) established that CRL3s are the main regulators of various cellular and developmental processes and stress responses. Working as an E3 ligase complex, conjugation of the ubiquitin-like protein Nedd8 to Cul3 is important for its function (Soucy et al., 2009). Lysine residue 712 near the C-terminus of Cul3 is necessary for the covalent modification of Nedd8 (Wimuttisuk and Singer, 2007).

Cul3 protein consists of 768 amino acid residues (Swiss-Prot: Q13618.2) in *Homo sapiens*. PHAII-causing mutations in *Cul3* may lead to the loss of exon 9 during splicing and generation of a mutant Cul3 protein with a 57-amino acid deletion (Δ403–459) (Boyden et al., 2012; Tsuji et al., 2013). In *Mus musculus*, Cul3 also comprises 768 amino acid residues (Swiss-Prot: Q9JLV5.1), and the genome sequence of exon 9 of *Cul3* is highly conserved between these species.

Patients with PHAII caused by *Cul3* mutations are heterozygous (Boyden et al., 2012; Tsuji et al., 2013). It is presumed that wild-type Cul3 is expressed in the kidneys of these patients, although it is unknown whether protein expression level is sufficient for its proper function. Furthermore, it is uncertain whether the skipping of exon 9 occurs or whether the deletion mutant is really present in the nephron segments where WNK and KLHL3 reside, or how mutations in these proteins cause kidney predominant PHAII phenotypes.

To answer these questions, we generated PHAII model mice carrying a mutation in *Cul3*. Of the many known disease-causing mutations, we selected two mutations: G(−1)A and T(−6)G. The splice acceptor site at the 3′ end of the intron is required for splicing; it contains an almost invariant AG sequence and terminates the intron. Both the mutations G(−1)A and T(−6)G were located in the 3′ end of intron 8 and were suspected to affect its splice acceptor function.

We successfully generated the following two knock-in mice: *Cul3*^G(−1)A/+^ and *Cul3*^T(−6)G/T(−6)G^. However, we were unable to demonstrate the loss of exon 9 during splicing in the *Cul3* knock-in mice. An examination of heterozygous *Cul3*^G(−1)A/+^ knock-in mice revealed that Cul3 expression level in the kidneys was reduced to approximately half of that in wild-type mice. This suggested that the mutant allele did not produce *Cul3* mRNA lacking exon 9, but rather behaved like a knockout allele in this mouse model. Furthermore, the analysis of *Cul3*^T(−6)G/T(−6)G^ knock-in mice revealed that Cul3 expression level from the mutant allele was modestly reduced in these mice.

This implied that, as expected, the guanine located at the 3′ end of intron 8 played an important role in the synthesis of normal mRNA. This single point mutation in the intron, but not in the exon, may have impaired the expression of intact Cul3 from the mutant allele in these mice. The mutated thymine may also have played a role in the synthesis of normal mRNA, but the mutation at this site may have been partially compensated.

When introducing the mutations, we selected the sites most conserved between *Homo sapiens* and *Mus musculus*. However, intron 8 was not completely identical between the species. Although we showed that the sites selected did play important roles in the synthesis of normal mRNA, differences in the overall sequences of the introns may have led to alternative consequences in terms of splicing abnormalities. Moreover, it may be possible that introduction of an FRT site disrupted an unknown enhancer located in the intron.

Next, we analyzed the effects of reduced Cul3 expression on WNK–OSR1/SPAK–NCC signaling. In our study, reduced Cul3 expression did not affect WNK–OSR1/SPAK–NCC signaling in *Cul3*^G(−1)A/+^ heterozygous knock-in mice or *Cul3*^T(−6)G/T(−6)G^ knock-in mice. Moreover, the mice did not have a phenotype resembling that of PHAII.

Wild-type Cul3 expression level had not been studied in the kidneys of patients carrying heterozygous PHAII-causing *Cul3* mutations. Based on our results in heterozygous knock-in mice, it is possible to speculate that the level of wild-type Cul3 expression may also be reduced in patients with PHAII, and that a reduction in Cul3 expression alone may be insufficient to cause the development of PHAII. This implies that mutant Cul3 protein has a more significant role in the pathogenesis of PHAII than decreased Cul3 expression levels. This may throw some light on why PHAII-causing mutations in *Cul3* cause kidney-predominant PHAII phenotypes.

Further studies including a study of PHAII model mice expressing the Cul3 mutant Δ403–459 are required to improve our comprehension of the pathogenic mechanisms of PHAII as related to *Cul3* mutations and the regulation of WNK–OSR1/SPAK–NCC signaling by Cul3. Also, analysis of the undefined functions of the Cul3 mutant Δ403–459 may improve our understanding of the function of Cul3.

## MATERIALS AND METHODS

### Generation of pseudohypoaldosteronism type II model mice carrying a mutation in *cullin 3*

To generate *Cul3* knock-in mice, we prepared a bacterial artificial chromosome clone (RPCI-23-81J23) containing the mouse genomic *Cul3* locus. Targeting vectors were prepared using polymerase chain reaction (PCR)-amplified segments of *Cul3* after verifying their sequences. The targeting vectors were then transfected into Baltha1 embryonic stem (ES) cells (Kim-Kaneyama et al., 2011) by electroporation, as described previously (Sohara et al., 2006). After selection with G418 and ganciclovir, correctly targeted ES cell clones were selected by PCR with the forward primers F1 (5′-TTATGTGGACAGCAAATGGTG-3′) and F2 (5′-TCGACTAGAGCTTGCGGAACCCTTC-3′) and the reverse primers R1 (5′-GCCAGAGGCCACTTGTGTAG-3′) and R2 (5′-CCTGGATCAGCTATACATATGCCTCTTGG-3′), and were verified by southern blotting and sequencing of the mutation site.

ES cells were injected into blastocysts to generate chimeric mice. Chimeric male offspring were mated with C57BL/6 females, and heterozygous mice were produced. The flippase recognition target (FRT)-flanked neomycin cassette was then deleted by crossing the heterozygous mice with FLPe recombinase-expressing transgenic mice (Farley et al., 2000). Genotyping of the mice was performed by PCR using the forward primer F3 (5′-AACCTGAGATGTCTGGAGGAC-3′) and the reverse primer R3 (5′-GATGTTGCCTGAACTCATCCAT-3′). The mice were raised in a 12-h day–night cycle, and fed a normal rodent diet and plain drinking water. The phenotype of the mice was evaluated at 8–10 weeks of age. This study was carried out in strict accordance with the recommendations of the Guide for the Care and Use of Laboratory Animals by the National Institutes of Health (Bethesda, MD, USA). The experiment was approved by the Animal Care and Use Committee of Tokyo Medical and Dental University, Tokyo, Japan. All efforts were made to minimize the suffering of the mice.

### Blood analysis and blood pressure measurements

Blood for electrolyte analysis was obtained as previously described (Yang et al., 2007). Electrolyte levels were determined using an i-STAT^®^ Portable Clinical Analyzer (Fuso Pharmaceutical Industries, Ltd., Osaka, Japan). Blood pressure in restrained conscious mice in a steady state was measured with a programmable tail-cuff sphygmomanometer (MK-2000A; Muromachi Kikai Co., Ltd., Tokyo, Japan).

### Immunoblotting

Semiquantitative immunoblotting was performed as previously described (Yang et al., 2007). For semiquantitative immunoblotting, we used either entire kidney samples without the nuclear fraction (600×***g***) or the crude membrane fraction (17,000×***g***). The relative band intensity of the immunoblots was analyzed and quantified using ImageJ software (National Institutes of Health). The primary antibodies used in this study were as follows: rabbit anti-Cul3 (1:1000, A301-109A; Bethyl Laboratories, Inc., Montgomery, TX, USA); rabbit anti-WNK1 (1:500, A310-531A; Bethyl Laboratories, Inc.); anti-WNK4 (1:250) (Ohta et al., 2009); rabbit anti-phosphorylated SPAK (1:500) (Sohara et al., 2011); rabbit anti-SPAK (1:250; Cell Signaling Technology, Inc., Danvers, MA, USA); anti-phosphorylated OSR1/SPAK (1:3000) (Ohta et al., 2009); anti-OSR1 (1:250, M10; Abnova Corporation, Taipei, Taiwan); rabbit anti-phosphorylated NCC (S71; 1:200) (Sandberg et al., 2007); guinea pig anti-NCC (1:200) (Isobe et al., 2013; Ohno et al., 2011); rabbit anti-actin (1:250; Cytoskeleton, Inc., Denver, CO, USA); rabbit anti-phosphorylated Na-K-Cl cotransporter isoform 2 (NKCC2; 1:300) (Yang et al., 2010); and guinea pig anti-NKCC2 (1:1000; provided by K. Mutig, Department of Anatomy, Charité-Universitätsmedizin, Berlin, Germany) (Mutig et al., 2007). Alkaline phosphatase-conjugated anti-immunoglobulin G antibodies (Promega Corporation, Madison, WI, USA) were used as secondary antibodies and Western Blue (Promega Corporation) was used to detect the signal.

### Splicing assay

We extracted RNA from peripheral blood lymphocytes using a Tempus Spin RNA Isolation Kit (Applied Biosystems Inc., Carlsbad, CA, USA) and from kidneys using TRIzol Reagent (Invitrogen, Carlsbad, CA, USA). mRNA was reverse-transcribed with random primers (Takara Bio Inc., Otsu, Shiga, Japan). A 664-base pair (bp) segment (493 bp, if exon 9 was skipped) was PCR-amplified from the cDNA using the forward primer F4 (5′-ACAGAAGACCTTGCTTGCATGT-3′) and the reverse primer R4 (5′-GCCAATATCCCGTTGTGAGAA-3′). The PCR products were fractionated and visualized via agarose gel electrophoresis and verified by sequencing.

### Quantitative polymerase chain reaction

Quantitative PCR was performed using SYBR Premix Ex Taq™ II (Takara Bio Inc.) and primers specific for *Cul3*, using the forward primer F4 and the reverse primer R5 (5′-AGCCCTGGATATAGTCAACAGG-3′). Gene expression analysis was performed by applying the ΔΔCt method and normalized against *β-actin*.

### Statistical analysis

Comparisons between the two groups were performed using unpaired *t*-tests. *P*-values <0.05 were considered statistically signiﬁcant. Data are presented as the means±standard error of the mean (s.e.m.).
